# Risikokommunikation politikberatender Wissenschaftsorganisationen: Ein Themenaufriss am Beispiel des Bundesinstituts für Risikobewertung

**DOI:** 10.1007/s00103-022-03520-3

**Published:** 2022-04-05

**Authors:** Fabian Brand, Leonie Dendler, Suzan Fiack, Annett Schulze, Gaby-Fleur Böl

**Affiliations:** Abteilung Risikokommunikation, Bundesinstitut für Risikobewertung (BfR), Max-Dohrn-Str. 8–10, 10589 Berlin, Deutschland

**Keywords:** Risikoanalyse, Regulierungswissenschaft, Partizipation, Health Literacy, Desinformation, Risk analysis, Regulatory science, Participation, Health literacy, Disinformation

## Abstract

Regulierungswissenschaftliche Organisationen wie das Bundesinstitut für Risikobewertung (BfR) sehen sich in ihrer wissenschaftsbasierten Risikokommunikation mit diversen Herausforderungen konfrontiert: Einerseits wird die Kommunikation gesundheitlicher Risiken immer komplexer und dementsprechend voraussetzungsreicher, weshalb unter anderem Fragen nach der Gesundheitskompetenz von Verbraucherinnen und Verbrauchern sowie zielgruppengerechter Risikokommunikation an Bedeutung gewinnen. Andererseits sehen sich die Wissensbestände regulierungswissenschaftlicher Organisationen zunehmend der Politisierung und öffentlichen Kritik ausgesetzt. In diesem Rahmen werden Fragen nach der Objektivität und Vertrauenswürdigkeit von Gutachten, Risikobewertungen und Stellungnahmen sowie der Legitimierung und Reputation regulierungswissenschaftlicher Organisationen relevant. Zusätzlich intensiviert wird dies durch das Aufkommen neuer Akteure in den sozialen Medien, die eigene Informations- und Kommunikationsmaterialien produzieren und veröffentlichen. In diesem Kontext verbreitete Fehl‑, Des- und Malinformationen stellen eine weitere Herausforderung dar, welche eng mit Fragen nach einer adäquaten Kommunikation über gesundheitliche Risiken sowie der Stabilisierung von Legitimität, Reputation und Vertrauenswürdigkeit zusammenhängt.

Der Artikel diskutiert verschiedene Lösungsansätze, darunter die Optimierung und visuelle Aufbereitung von Gesundheitsinformationen, die Ermöglichung gesellschaftlicher Partizipation und die Einbettung dieser Maßnahmen in das strategische Stakeholder- und Reputationsmanagement. Der Beitrag schließt mit einem Aufruf zu offenerer Diskussion inhärenter Dilemmata.

## Abstract (en)

Regulatory scientific agencies, such as the German Federal Institute for Risk Assessment (BfR), are confronted with various challenges in their science-based risk communication. On the one hand, the communication of health risks is becoming increasingly complex and, accordingly, more demanding, which is why – among other things – questions about the health literacy of consumers and target group-oriented risk communication are gaining importance. On the other hand, the outputs of regulatory scientific agencies are increasingly exposed to politicisation and public criticism, which increases the relevance of questions about the objectivity and trustworthiness of expert opinion, risk assessment, and official statements, as well as the legitimacy and reputation of such organisations. This is further intensified by the emergence of new social media actors who produce and publish their own information and communication materials. The misinformation, disinformation and malinformation consequently distributed in this context represent another challenge, which is closely related to questions of adequate communication of health risks and of stabilising legitimacy, reputation and trustworthiness.

The article discusses various approaches to solving these problems, including the optimization and visual enhancement of health information, enabling social participation as well as embedding these measures in strategic stakeholder and reputation management. The article concludes with a call for a more open discussion of inherent dilemmas.

## Einleitung

Die COVID-19-Pandemie (englisches Akronym für: *Coronavirus Disease 2019*) hat die wissenschaftsbasierte Risikokommunikation vor neue Herausforderungen gestellt. Dem *Wissenschaftsbarometer 2020 *[1] zufolge ist das Vertrauen der deutschen Bevölkerung in Wissenschaft und Forschung zwar höher als in den Jahren zuvor, wohl aber ist der Anteil der Befragten, die angaben, Wissenschaft und Forschung eher oder voll und ganz zu vertrauen, von anfänglich 73 % im April 2020 auf zwischenzeitlich 60 % im Dezember 2020 gefallen. Ähnliches zeigt sich in vielen anderen Ländern [2]. Somit wird deutlich, wie volatil der Vertrauensvorsprung, welcher wissenschaftsbasierter Risikokommunikation zugeschrieben wird, sein kann. Diverse Herausforderungen stellen sich hier besonders für Organisationen wie das Bundesinstitut für Risikobewertung (BfR), dessen Aufgabe es ist, auf Basis wissenschaftlicher Stellungnahmen – zum Beispiel im Zusammenhang mit Viruskontaminationen auf Lebensmitteln und Oberflächen – politische Entscheidungsträger zu beraten und die Öffentlichkeit zu informieren.

So werden einerseits Risikozusammenhänge zum Beispiel aufgrund globaler Waren- und Produktionsketten, aber auch aufgrund veränderter Konsumtionsmuster immer komplexer, weshalb auch die Kommunikation solcher Risiken diffiziler wird. Zum anderen werden die Informationsdichte und -heterogenität zu einer Herausforderung für die Risikokommunikation [3]. Wissenschaftliche Studien, Gutachten oder Stellungnahmen werden in ihren Schlussfolgerungen auch dadurch rechtfertigungsbedürftig. Dies gilt ganz besonders für Organisationen wie das BfR, die sich in kontroversen Feldern wie dem der Ernährung bewegen [4, 5]. Ernährung bedeutete im Lauf der Menschheitsgeschichte nie nur die reine Aufnahme von Nahrungsmitteln, sondern ist vielmehr zu einem Gegenstand des Genusses und Erlebens, des Wissens und der Kompetenzen, der Performanz und des sozialen Prestiges, aber auch der Sorge avanciert [6]. Die sich daraus ergebende Konsumentennähe zu Lebensmittelprodukten wird konterkariert von einer zunehmenden Globalisierung und Komplexität in der Produktion von Lebensmitteln [7]. So wurde die globale Lebensmittelproduktion das Subjekt zahlreicher Krisen, die nicht nur punktuell Produkte, sondern gesamte Vertrauensbildungsprozesse infrage stellten.

In der Tat kann bereits die Gründung des BfR als eine Herausforderung bezeichnet werden. Diese fand im Rahmen einer fundamentalen europaweiten Neuordnung des Lebensmittelsicherheitssystems statt, die das im Kontext der BSE-Krise^1^ geschwächte öffentliche Vertrauen durch eine klarere Trennung von Politik und Wissenschaft sowie eine ausdrücklichere Fokussierung auf Verbraucherinteressen stärken sollte [8, 9]. Gegründet als unabhängige regulierungswissenschaftliche Organisation^2^ im Geschäftsbereich des Bundesministeriums für Ernährung und Landwirtschaft (BMEL) sind die Hauptaufgaben des BfR die Bewertung bestehender und das Erkennen neuer gesundheitlicher Risiken in den Bereichen Lebensmittel‑, Produkt- und Chemikaliensicherheit, die Erarbeitung von Empfehlungen zur Risikobegrenzung sowie die transparente Kommunikation dieses Prozesses. Um diese Tätigkeiten zu unterstützen, betreibt das BfR auch eigene sozialwissenschaftliche Forschung auf den Gebieten der Risikokommunikation und der Risikowahrnehmung.

Die Einrichtung regulierungswissenschaftlicher Organisationen steht im engen Zusammenhang mit einer Neuordnung entlang des sogenannten Red-Book-Modells des United States National Research Council (NRC). In der vorherrschenden Auslegung dieses Modells sollen politische Entscheidungen, zum Beispiel im Bereich der Lebensmittelsicherheit, durch wissenschaftliche Bewertungen informiert werden, die wiederum frei von politischen Einflüssen verfasst wurden [8, 11, 12]. So soll das BfR „durch eine klare organisatorische Trennung von den politisch geprägten Strukturen des Risikomanagements … frei von äußerer Einflussnahme und unabhängig sein“ [13]. In der Praxis sieht sich regulatorisches Wissen [10] jedoch mit einer Vielzahl an Erwartungen und Zieldefinitionen konfrontiert. Für die Risikokommunikation ergeben sich daraus verschiedene Herausforderungen – nicht nur, weil die Risikowahrnehmung von Bürgerinnen und Bürgern durch psychologische, soziale und kulturelle Faktoren [14] sowie politische Präferenzen [15] konturiert wird und Einstellungen gegenüber bestimmten Organisationen die Wahrnehmung spezifischer Risiken beeinflussen können [16]. Im öffentlichen Diskurs artikulieren sich zudem immer wieder politische Ansprüche, die auch unmittelbar an Organisationen wie das BfR adressiert werden – trotz des institutionellen Rahmens, welcher das BfR von politischen Ansprüchen immunisieren soll [5]. Vor diesem Hintergrund skizziert dieser Beitrag anhand des Beispiels des BfR praktische Herausforderungen für die wissenschaftsbasierte Risikokommunikation und diskutiert mögliche Lösungsansätze sowie Forschungsbedarfe.

## Herausforderungen wissenschaftsbasierter Risikokommunikation

### Risikowahrnehmung und Gesundheitskompetenz

Die COVID-19-Pandemie markiert die Herausforderungen effektiver Gesundheitskommunikation in besonders dramatischer Weise. Nicht nur scheint die Bereitschaft zu gesundheitspräventiven Maßnahmen durch die Risikowahrnehmung beeinflusst zu sein [17], sondern zugleich betonen einzelne Studien Probleme bei der Einordnung, dem Verstehen sowie der Nutzung von COVID-19-relevanten Informationen in großen Teilen der Bevölkerung [18] – und verweisen dementsprechend auf Fragen der Gesundheitskompetenz.

Für regulierungswissenschaftliche Einrichtungen wie das BfR stellt sich das Problem der Gesundheitskompetenz bestimmter Zielgruppen in besonderem Maße. Gesundheitskompetenz bzw. *Health Literacy*, also die Möglichkeit, Gesundheitsinformationen zu finden, zu verstehen und zu nutzen [19], spielt gerade im Lebensmittelbereich eine besondere Rolle, wirkt sich diese doch beispielsweise auf das individuelle Ernährungsverhalten aus. Dabei ist schon die verständliche Kommunikation von *Risiken* – in Abgrenzung von *Gefahren – *herausfordernd. Das Allgemeine Lebensmittelgesetz definiert die Begriffe *Gefahr *bzw. *Gefährdungspotenzial *als Agens in oder Zustand von Lebens- oder Futtermitteln, das oder der eine Gesundheitsbeeinträchtigung verursachen kann [20]. Der Begriff des *Risikos *fokussiert auf den eigentlichen Kontakt eines Menschen mit einem gefährlichen Stoff, d. h. die Menge des Stoffes sowie die Art des Kontaktes selbst. In der Risikoanalyse und Toxikologie bezeichnet der Begriff des Risikos somit das Zusammenspiel von Gefährdungspotenzial und Exposition.

Während einige Akteure also strikt zwischen der Gefahr und dem Risiko eines Stoffes differenzieren, zeigte eine vom BfR in Auftrag gegebene Studie, dass diese Begriffe nicht nur in verschiedenen Wissenschaftsdisziplinen unterschiedlich gebraucht werden, sondern auch, dass verschiedene Akteursgruppen mit unterschiedlichen Risikokonzeptionen operieren [21]. Für die Risikokommunikation besteht aufgrund der divergierenden Begriffsbestimmungen und Verwendungen von *Risiko* und *Gefahr* [22] somit nicht nur ein theoretisches Problem, denn deren fehlende Unterscheidung hat in den letzten Jahren wiederholt zu öffentlichen Kontroversen geführt. So stufte die Internationale Agentur für Krebsforschung (IARC) der Weltgesundheitsorganisation (WHO) im Jahr 2015 verarbeitetes Fleisch als Karzinogen der Gruppe 1, also als krebserzeugend, ein [23]. In dieser Gruppe befinden sich auch Asbest oder Tabakrauch. Die Einstufung in Gruppe 1 trifft jedoch keine Aussage über das tatsächliche, mit den Substanzen verbundene Krebsrisiko. Die IARC führt eine rein gefahrenbezogene Analyse durch, die gemäß der Präambel keine Empfehlungen für Regierungen und Behörden darstellen soll. Konträr hierzu nimmt das BfR eine risikobezogene Bewertung vor. Diese berücksichtigt neben der gefahrenbezogenen Analyse eines Stoffes auch die geschätzte Exposition, also die tatsächliche Aufnahmemenge des Stoffes.

**Figure 1:**
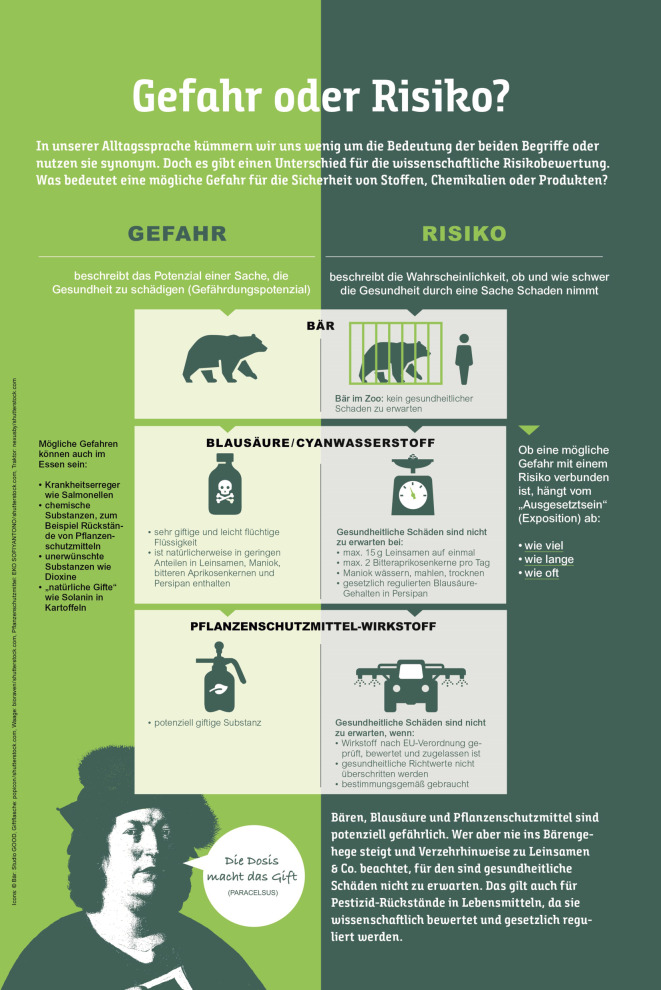

Das komplexe Verhältnis zwischen *Gefahr *und *Risiko*^3^ fordert die wissenschaftsbasierte Risikokommunikation also immer wieder heraus, zumal Verbraucherinnen und Verbraucher weniger durch das mögliche Risiko als vielmehr durch das bloße Vorhandensein einer chemischen Substanz in Lebensmitteln beunruhigt zu sein scheinen [26]. Um ein vertieftes Verständnis von Risikobewertungszusammenhängen zu fördern, bietet sich beispielsweise die Nutzung spezifischer Visualisierungsformen an. Zur Unterstützung von Verständnis, Wahrnehmung und Entscheidungsfindung, aber auch von sozialer Akzeptanz und Vertrauen [27] wurde wiederholt auf die Relevanz des Einsatzes von Diagrammen, visuellen Metaphern, grafisch unterstützten Erläuterungen oder Karten für die Risikokommunikation verwiesen [28]. Informationsvisualisierungen können die Entscheidungsqualität und Urteilsgenauigkeit sowie das Verständnis von Wahrscheinlichkeiten und (wissenschaftlicher) Unsicherheit unterstützen [29]. Dieser Argumentation folgend entwickelte das BfR eine Grafik, in welcher der terminologische Unterschied zwischen *Gefahr* und *Risiko* anhand verschiedener Gefährdungspotenziale (Bären, Blausäure und Pflanzenschutzmitteln) durch Symbole, Icons und Textinhalte erklärt wird (Abb. 1). Die Grafik wird derzeit in Zusammenarbeit mit der European Food Safety Authority (EFSA) erweitert.

Dabei zeigt sich ein inhärentes Dilemma solcher Visualisierungen: Zwar können sie zur Verbesserung von Risikoverständnis und -wahrnehmung beitragen, setzen jedoch die Fähigkeit voraus, zahlenbasierte wie auch visuell aufbereitete Risikoinformationen einordnen und verstehen zu können [31]. Visualisierungen können „komplexe Sachverhalte in schematischer Weise darstellen, so dass sachlogische Zusammenhänge, die in sprachlicher Form nur bedingt nachvollziehbar sind, anschaulich und intuitiv einsehbar werden“ [32]. Auf Seite der Rezipientinnen und Rezipienten setzen sie dann jedoch die Kompetenz voraus, visuelle Codes erkennen und interpretieren zu können. Mit der zunehmenden Relevanz von Visualisierungen avanciert das Beherrschen der visuell-numerischen Sprache (*Graphicacy*) zu den „Schlüsselkompetenzen für das Kommunizieren und Verstehen von politischen, sozialen und kulturellen Phänomenen“ [33]. Dabei stellt sich zunehmend auch für die Risikokommunikation die Frage, wie Informationen adäquat aufbereitet werden können, um nicht nur zu informieren, sondern auch ein entsprechendes Präventionsverhalten zu evozieren.

Eine mögliche Antwort ist die konsequente Anwendung gesundheitstheoretischer Modelle und Erkenntnisse aus Wissenschaftsdisziplinen wie der Kommunikationswissenschaft, der Psychologie und der Soziologie: Wie risikobezogene Informationen aufbereitet und zielgruppengerecht kommuniziert werden und wie Verbraucherinnen und Verbraucher Kommunikation interpretieren, lässt sich über theorie- und empiriegeleitete Forschung analytisch erfassen [34]. Die entsprechenden Befunde ermöglichen dann die Entwicklung zielgruppenspezifischer Formate und Inhalte.

Gleichzeitig werden mit der Frage nach einer (visuell) adäquaten Aufbereitung von Gesundheitsinformationen auch substanziellere Fragen tangiert. Sozialpsychologische Studien haben beispielsweise wiederholt auf kognitive Phänomene wie *Motivated Reasoning *verwiesen. *Motivated Reasoning *spielt dabei insbesondere auf die selektive Interpretation und Verarbeitung von Informationen entsprechend bestehender Dispositionen und Überzeugungen der Rezipientinnen und Rezipienten an [35]. Zur Adressierung solcher Herausforderungen werden in der wissenschaftlichen Literatur vermehrt auch *partizipativere Formen *der Wissenschaftskommunikation diskutiert, in der die Zielgruppen beispielsweise aktiv in die Entwicklung und Distribution von Kommunikationsmaßnahmen involviert werden [36]. Hierdurch soll unter anderem^4^ die Effizienz der übermittelten Informationen verbessert, Vertrauen gestärkt [37] und eine bessere Einbettung in die heterogenen Lebenswelten der diversen Zielgruppen erreicht werden, um damit die Effektivität von Kommunikationsmaßnahmen zu erhöhen. Indem ein „intrinsisches“ Verständnis für die Informationsbedürfnisse der Zielgruppen entwickelt wird [38], können die verschiedenen Risikoinformationen eine stärkere Verbreitung und Verankerung in der Bevölkerung finden [39]. Die Zielgruppen werden nicht mehr nur als Rezipientinnen und Rezipienten verstanden, sondern vielmehr in alle Schritte des Wissensproduktionsprozesses und der Entwicklung kommunikativer Interventionen einbezogen [40]. Die Forschungen auf diesem Feld stellen ein wichtiges Desiderat für die Risikokommunikation dar.

### Pluralisierung und Politisierung von Wissen

Neben der Frage nach der Risikowahrnehmung, der Gesundheitskompetenz spezifischer Bevölkerungsgruppen und der Rezeption und Produktion von Risikokommunikationen hat die COVID-19-Pandemie auch Fragen nach der Politisierung von Wissen neu aufgeworfen. Politische Streitfragen – etwa um die Angemessenheit von gesundheitspräventiven Maßnahmen – bekommen heute „immer stärker epistemischen Charakter, werden zu Wissenskonflikten“, weil sich die Komplexität gesellschaftlicher Problem- und Risikolagen nicht mehr so einfach „in eine relativ übersichtliche politische Interessen- oder Verteilungsfrage übertragen lässt“ [41].

Gerade im Bereich der Ernährung wird Vertrauen dabei unterschiedlich konstruiert. Während die einen den Fokus auf Lebensmittelsicherheit in Form einer Quantifizierung von Risiken und Standardisierung von Wertschöpfungsketten legen, stellen andere ethische Aspekte wie Umwelt- oder Tierschutz in den Vordergrund. Im Rahmen einer Polarisierung von Lebensmitteleigenschaften – global vs. regional, synthetisch vs. authentisch, chemisch vs. natürlich – wird beispielsweise das *Natürliche* häufig diskursiv als *gut* gerahmt und synthetische Chemikalien wie Pflanzenschutzmittel hingegen oft als *schlecht* [42, 43]. In der Tat führen Rückstände von Pflanzenschutzmitteln in Lebensmitteln bei gestützten Bevölkerungsabfragen regelmäßig die Liste der Themen an, von denen angenommen wird, dass sie die Qualität und Sicherheit von Lebensmitteln beeinträchtigen können [44, 45].

Die Kommunikation wissenschaftlicher Erkenntnisse muss daher in ihrer sozialen Kontextualisierung gedacht werden. Dabei werden sowohl die Ergebnisse regulierungswissenschaftlicher Arbeit – zum Beispiel die konkrete Bewertung eines Risikos – als auch die dahinterliegenden Wissenschaftskonzepte und -verfahren kontrovers diskutiert [10]. So kritisierten einzelne Akteure im Rahmen der durch das BfR erfolgten Bewertung des gesundheitlichen Risikos von Glyphosat die Studienbasis sowie eine mangelnde Objektivität und Unabhängigkeit des BfR. Darüber hinaus wurde die Bewertung wiederholt zum Anlass genommen, grundlegende Reformen in der Bewertung von Lebensmittelrisiken sowie der Lebensmittelproduktion im Allgemeinen zu fordern [46].

Damit wird deutlich, dass sich die Produzentinnen und Produzenten wissenschaftlicher Risikobewertungen immer auch der öffentlichen Kritik, Politisierung sowie der diskursiven Mobilisierung durch andere gesellschaftliche Akteure stellen müssen. Innerhalb dieses Prozesses werden nicht nur gesamtgesellschaftliche Fragen verhandelt, die oft weit über die administrativen Zuständigkeiten einzelner Organisationen hinausgehen, sondern auch verschiedene Logiken der Wissensgenerierung und Legitimierung konstruiert, die zumindest in Teilen miteinander in Konflikt stehen [8, 47]. Besonders deutlich lässt sich dies an der Kategorie der Objektivität illustrieren. Wie das oben genannte Beispiel verdeutlicht, bildet Objektivität nicht nur einen von einigen Akteuren postulierten Standard wissenschaftlicher Forschung, sondern fungiert regelmäßig als kommunikative Ressource, die mobilisiert und reputations- oder legitimitätsschädigend eingesetzt werden kann [48]. Während Objektivität von vielen Akteuren im Sinne einer *Proper Representation of Nature* (dt.: angemessenen Abbildung der Natur) und als Teil der Wertfreiheit und Unabhängigkeit von Wissenschaft gesehen wird [49], weisen andere darauf hin, dass in die „scheinbare Eindeutigkeit, Richtigkeit, Neutralität und Interessenlosigkeit“ von Verfahren der Wissensgenerierung immer auch Machtkonflikte und unterschiedliche Machtpositionen eingelassen sind [47]. Viele Autorinnen und Autoren stellen dabei die grundsätzliche Möglichkeit wertfreier wissenschaftlicher Verfahren infrage. Vielmehr sehen sie unausgesprochene Bestrebungen, Werturteile und Grundannahmen als versteckte Faktoren in der Konstruktion von Wissen [12, 50]. Wissen, welches die Standards der dominanten Wissensproduktion nicht erfüllt, kann hierbei marginalisiert werden [10, 51].

Vor diesem Hintergrund finden sich immer mehr Bestrebungen nach stärkerer Transparenz und einer Demokratisierung der (Regulierungs‑)Wissenschaft. In der Europäischen Union (EU) ist im Jahr 2021 beispielsweise die *Verordnung über die Transparenz und Nachhaltigkeit der EU-Risikobewertung im Bereich der Lebensmittelkette* [52] in Kraft getreten. Unter anderem erhalten Bürgerinnen und Bürger Zugang zu Studien und Informationen, die die Industrie im Verlauf einer Risikobewertung vorlegt. Zudem werden Stakeholder und Öffentlichkeit zu den vorgelegten Studien konsultiert. Die Berücksichtigung breiterer gesellschaftlicher Perspektiven soll nicht nur die Transparenz von Forschungs- und Risikobewertungsprozessen verbessern und gesellschaftliche Unterstützung sichern, sondern auch die Komplexität und Robustheit von Evidenzen garantieren [3]. Die (prozedurale) Inklusion weiterer Perspektiven fungiert als Mittel, das epistemische Risiko einer (absichtlichen oder unabsichtlichen) Verzerrung von Ergebnissen zu reduzieren und damit eine sich aus der Kollektivität ergebende Objektivität sicherzustellen [53]. Dies konfligiert jedoch notwendigerweise mit der oben beschriebenen Unabhängigkeitserwartung anderer Akteure und führt zu einem Dilemma, welches mit der Objektivität regulierungswissenschaftlichen Wissens korrespondiert: Während für die einen Objektivität durch eine gewisse, von Werten und Interessen abstrahierende Distanz, d. h. mithin durch die Unabhängigkeit regulierungswissenschaftlicher Organisationen, garantiert werden soll, kann für die anderen die Berücksichtigung weiterer Perspektiven überhaupt erst – aus epistemischen wie nichtepistemischen Gründen – Objektivität garantieren [54].

Diese Herausforderungen zeigten sich auch in einer Studie des BfR zur partizipativen Öffnung von Risikobewertungsorganisationen auf Basis von Stakeholder- und Bevölkerungsbefragungen [8]. Zwar wurde eine generelle Unterstützung für eine partizipative Öffnung des Risikobewertungsprozesses verbalisiert, diese basiert jedoch primär auf Argumenten der Evidenzerweiterung. Die potenzielle Stärkung gesellschaftlichen Vertrauens in regulierungswissenschaftliche Organisationen und ihre Wissensobjekte wurde – zusammen mit ungleich verteilten Partizipationskapazitäten – eher kritisch diskutiert. Dies gilt insbesondere bezüglich einer tiefgehenden Öffnung regulierungswissenschaftlicher Entscheidungsprozesse (beispielsweise für Stakeholderbeiräte), in der einige Befragte eine Gefährdung für die wissenschaftliche Unabhängigkeit und damit die Reputation des BfR sahen.

Eine positive Reputation kann die Deutungshoheit und Autorität von Organisationen wie dem BfR unterstützen und Vertrauen stärken [55]. Die obigen Ausführungen machen dabei deutlich, dass strategisches Reputations- und Stakeholdermanagement immer auch ein Balanceakt zwischen konfligierenden Ansprüchen darstellt. Hierbei müssen wichtige Faktoren wie (wissenschaftliche) Leistung oder technische und wissenschaftliche Kompetenz adressiert, zugleich jedoch auch die diskursiv konstruierte Wahrnehmung oder auch Nichtwahrnehmung dieser Faktoren berücksichtigt werden [56]. Letztere Aspekte finden in der einschlägigen Literatur bisher nur begrenzt Beachtung und bilden einen weiteren wichtigen Forschungsbereich für das BfR.

### Ausdifferenzierung von Sprecherrollen: Soziale Medien, Prosumer und Desinformationen

Mit dem Aufkommen des Internets und den damit verbundenen Beteiligungsmöglichkeiten intensivieren sich die oben skizzierten Herausforderungen. Im Rahmen eines „digitalen Wandel[s] der Wissensordnung“ [57] hat sich die Produktion, Distribution und Nutzung von Kommunikationsinhalten verändert [58]: Beobachten lassen sich eine Pluralisierung von Öffentlichkeit, die Entstehung unterschiedlicher Teilpublika sowie eine Ausdifferenzierung von Sprecherrollen. Zunehmend treten sogenannte Prosumer [59] – also Akteure, die Kommunikationsmaterialien nicht nur rezipieren, sondern diese beispielsweise deuten, reformulieren oder rekontextualisieren – mit eigenen Kommunikationsangeboten auf die Bühne. Dabei werden vermehrt auch Fehl‑, Des- und Malinformationen verbreitet [60, 61], die jedoch nicht immer auf dezidiert wissenschafts*feindliche *Narrative rekurrieren, sondern vielmehr ebenfalls auf wissenschaftliche Semantiken zurückgreifen und dabei auf die vermeintliche Evidenzbasierung und Faktizität ihrer Inhalte verweisen [62].

Für regulierungswissenschaftliche Organisationen kann diese zunehmende Distribution von Fehl‑, Des- und Malinformationen über soziale Medien wie *Facebook *oder *Twitter*, aber auch das *Dark Social *– also beispielsweise Messengerdienste wie *Whatsapp oder Telegram –* besonders problematisch werden. Die COVID-19-Pandemie hat dies in eindrücklicher Weise vorgeführt [63]. Die Emergenz neuer Kommunikationstechnologien hat neue Produktions‑, Diffusions- und Nutzungspraktiken hervorgebracht [58] und führt nicht nur quantitativ zu einer Proliferation von gesundheitsrelevanten Informationsmaterialien, sondern erschwert auch zunehmend die Beurteilung der Informationsqualität. Die Häufung von *Infodemien *[64], in welcher ein Übermaß an Informationen die Identifikation und Bewertung der Quellen beeinträchtigt, stellt auch das BfR vor Herausforderungen: gesundheitspräventiv aufgrund der Schwierigkeiten, mit denen Verbraucherinnen und Verbraucher bei der Identifizierung von Falschinformationen konfrontiert sind [65] und diese Falschinformationen sich immer auch negativ auf deren individuelle Gesundheit auswirken können [66]; strategisch-kommunikativ, weil etwa Personen mit einem hohen Vertrauen in *Wissenschaft *zugleich auch besonders anfällig für *pseudowissenschaftliche *Informationsangebote zu sein scheinen [67] und Fehlinformationen sich online stärker und schneller verbreiten als faktenbasierte, (wissenschaftlich) gesicherte Informationen [68].

Gleichwohl muss die Definition *richtiger* Informationen kritisch reflektiert und diskutiert werden [69]. Hierbei ist unter anderem zu berücksichtigen, dass wissenschaftliche Unsicherheit Teil wissenschaftlichen Forschens ist und Erkenntnisse sich aufgrund neuer Daten ändern. Demnach sind auch regulierungswissenschaftliche Organisationen darin herausgefordert, den Wissensstand so zu kommunizieren, dass Verbraucherinnen und Verbraucher die Gültigkeit auf Zeit nachvollziehen können. Denn nur insofern ein Verständnis für die Funktionsweisen von Wissenschaft vorhanden ist, kann der Unterschied zu falschen Informationen, die unabsichtlich (Fehlinformation) oder absichtlich (Desinformation) veröffentlicht werden, wahrgenommen werden [61].

Besonders herausfordernd ist dabei der Umstand, dass die Geschwindigkeit, mit der Falschinformationen produziert, aber auch distribuiert werden können, häufig mit der Systemzeit der Wissenschaft konfligiert, ist der wissenschaftliche Forschungsprozess doch von Verifizierungsschleifen sowie der Notwendigkeit, Ergebnisse evidenzbasiert rechtfertigen zu können, gekennzeichnet. Wissenschaftsbasierte Risikokommunikation muss Aspekte wie wissenschaftliche Unsicherheiten berücksichtigen und entsprechend adressieren – ein Erfordernis, welches dem Bedürfnis nach einfachen, schnell verständlichen Informationen mitunter zuwiderläuft. Die wissenschaftsbasierte Risikokommunikation steht vor der Aufgabe, einen adäquaten Umgang mit den Informationsbedürfnissen der verschiedenen Zielgruppen (siehe Abschnitt zur Herausforderung „Risikowahrnehmung und Gesundheitskompetenz“) innerhalb eines zunehmend digitalen und schnelllebigen Kontextes zu finden, wobei eine unterkomplexe, an Einfachheit und Schnelligkeit orientierte Risikokommunikation von bestimmten Zielgruppen als unglaubwürdig wahrgenommen und Fragen nach ihrer Legitimität evozieren kann (siehe Abschnitt zur Herausforderung „Pluralisierung und Politisierung von Wissen“).

Die detailliertere Beleuchtung dieser Herausforderung und die potenzielle Relevanz von Social-Media-Monitoring bilden weitere wichtige Forschungsbereiche für die Risikokommunikation des BfR und anderer regulierungswissenschaftlicher Organisationen.

## Fazit

Dieser Artikel stellt die komplexen Herausforderungen, mit denen sich die Risikokommunikation des BfR heute konfrontiert sieht, dar. Dabei wurde einerseits die Frage nach der Risikowahrnehmung und der Gesundheitskompetenz der diversen Zielgruppen des BfR berührt: Wie kann adäquat über gesundheitliche Risiken kommuniziert werden, ohne dabei die Adressatinnen und Adressaten solcher Informationsbestrebungen aus dem Blick zu verlieren, sondern vielmehr deren Akzeptanz, Vertrauen und Verständnis fördern? Es wurde zudem auf ein zweites Problem hingewiesen, welches unmittelbar mit der Position des BfR an der Grenze von Wissenschaft und Politik zusammenhängt: Regulationswissenschaftliches Wissen ist in Zeiten der Politisierung und Pluralisierung rechtfertigungsbedürftig und muss u. a. seine (öffentlich mitunter angefochtene) Legitimierung immer wieder restabilisieren und behaupten. Mit der zunehmenden Relevanz sozialer Medien, der Ausdifferenzierung von Sprecherrollen und dem Auftreten neuer Akteure im Internet (*Prosumer*) werden diese Probleme dabei nochmals intensiviert, da eine Balance zwischen der Komplexität wissenschaftlichen Wissens und dessen Produktionsbedingungen sowie den Aufmerksamkeits- und Informationsbedürfnissen der diversen Zielgruppen gefunden werden muss.

Zur Lösung dieser Metaprobleme wurden zahlreiche Ansätze diskutiert: Von der Optimierung und visuellen Aufbereitung von Gesundheitsinformationen über die Ermöglichung von (zivilgesellschaftlicher) Partizipation bis hin zur Einbettung solcher prozessualen Anpassungen in das Stakeholder- und Reputationsmanagement. Dabei zeigte sich, dass keiner dieser Ansätze ein „Universalmittel“ darstellt, sondern diese Ansätze vielmehr inhärente Dilemmata aufweisen, die beständig austariert werden müssen. Hierzu ist es wichtig, sich der komplexen Einbettung regulierungswissenschaftlicher Organisationen bewusst zu werden und dabei die Heterogenität und partielle Widersprüchlichkeit spezifischer gesellschaftlicher Ansprüche zu berücksichtigen und offen zu diskutieren.

## Footnotes

1 BSE – umgangssprachlich auch als „Rinderwahnsinn“ bezeichnet – ist ein Akronym für die Rinderkrankheit *bovine spongiforme Enzephalopathie*, an der zunächst Rinder erkranken, deren Erreger aber auf den Menschen und andere Tiere übertragbar sind.

2 Der Begriff der *regulierungswissenschaftlichen Organisation* bezieht sich auf den Begriff des Regulierungswissens, also des Resultats bzw. Outputs von *Regulatory Science*: „Regulatory Science covers the testing of technologies and their risks and the interpretation of test results in a mixed industrial, bureaucratic and academic environment, to legitimize the adoption of policy measures“ [10].

3 Die Differenzierung der beiden Termini *Risiko *und *Gefahr* ist interdisziplinär durchaus heterogen. Für eine eher sozialwissenschaftlich orientierte Differenzierung, die eng mit der Unterscheidung von Entscheidern und Betroffenen korrespondiert, siehe etwa die klassischen Definitionen bei Luhmann [24] und Beck [25].

4 Weitere Argumente für partizipative Öffnungen werden im Abschnitt „Pluralisierung und Politisierung von Wissen“ diskutiert.
